# Questionable Effects of Electronic Cigarette Use on Cardiovascular Diseases From the National Health Interview Survey (NHIS, 2014-2021)

**DOI:** 10.7759/cureus.57119

**Published:** 2024-03-28

**Authors:** Nantaporn Plurphanswat, Arielle Selya, Brad Rodu

**Affiliations:** 1 Brown Cancer Center, University of Louisville, Louisville, USA; 2 Tobacco Harm Reduction, Pinney Associates Inc., Pittsburgh, USA

**Keywords:** cardiovascular diseases, myocardial infarction, national health interview survey, electronic cigarette, stroke

## Abstract

Background: Electronic cigarettes (e-cigarettes) and cardiovascular health risks have gained attention among tobacco researchers. While the cardiovascular risks from e-cigarettes are still unclear, a recent paper by Alzahrani in Cureus claimed that current usage of e-cigarettes increases the risks of cardiovascular diseases, such as myocardial infarction and stroke, in subjects who were never cigarette smokers.

Methods: The National Health Interview Survey (NHIS) data from 2014 to 2021 and logistic regression models were used to replicate and extend Alzahrani’s analysis.

Results: Only 12 never smokers who were current e-cigarette users had a myocardial infarction in all eight years. The crude odds ratio (OR) for e-cigarette use was 0.42 (95%CI: 0.24, 0.75). After adjusting for age and other confounding factors and health conditions, the OR of e-cigarette use increased to 2.48 (95%CI: 1.35, 4.55). The omission of age while adjusting for all other risk factors resulted in an OR of 0.80 (95%CI: 0.45, 1.43). In addition, the adjusted ORs for coronary heart disease and stroke were 1.12 (95%CI: 0.58, 2.17) and 1.13 (95%CI: 0.55, 2.29), respectively.

Conclusions: The findings indicate that Alzahrani’s study is scientifically unreliable. The association between e-cigarette use and heart attack reported by Alzahrani was substantially driven by age, and the very small number of exposed cases makes the association very unstable. Given the nature of cross-sectional NHIS data, it is impossible to establish a robust association or causal claim that e-cigarette use “increases” the risks of any disease.

## Introduction

Using National Health Interview Survey (NHIS) data from 2014 to 2021, Alzahrani [1] found that there is a positive association between electronic cigarette use and heart attack events among never smokers. Since the author published his study in 2023, many have raised issues with this study especially since he had implied a causal relationship between e-cigarette use and heart attack [2]. In March 2024, Alzahrani made a revision to the Abstract section stating that “current e-cigarette use may increase the risk of myocardial infarction…”, and a similar statement was used in the Discussion and Conclusion sections [3].

While the causal statements have been revised, there are a number of flaws, inaccurate statements, and inconsistencies in the article that we examine further in the current replication study.

Our specific aims are to replicate Alzahrani's [1] analysis and to examine related outcomes for the purpose of assessing the plausibility of his findings. Specifically, if his conclusions that e-cigarette use is associated with a higher risk of myocardial infarction are valid, then it would be expected that there would also be associations between e-cigarette use and related cardiovascular outcomes, including coronary heart disease and stroke.

Using the same NHIS data and statistical methods, we demonstrate that e-cigarette use is not associated with coronary heart disease and stroke, and we document that the statistically significant association between e-cigarette use and heart attack in the Alzahrani's analysis [1] stemmed from spurious correlations caused by confounding by age and the very small sample size of the exposed group.

## Materials and methods

Data

We obtained 2014-2021 NHIS data through the Integrated Public Use Microdata Series (IPUMS) Health Surveys: National Health Interview Survey (NHIS) [4]. Following Alzahrani's methods [1], the study population included participants who had never smoked at least 100 cigarettes in their lifetime, i.e., never smokers.

The outcomes of our study included self-reported heart attack, coronary heart disease, and stroke. Each outcome is derived from the following three questions: “Have you ever been told by a doctor or other health professional that you had … (1) a heart attack, also called myocardial infarction, (2) coronary heart disease, and (3) a stroke?”.

We used Alzahrani’s definitions of current (used every day or some days) and never e-cigarette users. Notably, participants who had never used e-cigarettes and did not use them at the time of the surveys were completely excluded by Alzahrani [1] from the analysis. We classify them as “former triers.”

We also incorporate other demographics (age and sex) and self-reported health conditions (diabetes, high blood pressure, high cholesterol overweight/obese, BMI ≥ 25) used by Alzahrani [1]. Notably, Alzahrani added age as a continuous variable; while this did not alter the ORs for e-cigarette use compared to using age groups (see below), it is inappropriate and not biologically meaningful [5] because it assumes that an increase in one year of age has the same association with heart attack throughout the age spectrum. Alzahrani should have included age squared in the model or used age groups as the risk of heart attack rises more rapidly at higher ages. We employed the latter approach using appropriate age groups (18-24, 25-34, 35-44, 45-54, 55-64, and 65+) to obtain a meaningful and interpretable age variable.

In addition, we added a categorical race/ethnicity variable (non-Hispanic White, non-Hispanic Black, Hispanic, and other races) and levels of education (less than high school, high school, some college, college or more) since cardiovascular disease prevalence varies by race/ethnicity [6] and levels of education [7,8].

Furthermore, previous studies have suggested that heart disease mortality varies by geography and years [9-11], so we included categorical variables for U.S. regions (Northeast, Midwest, South, West) and years (2014, 2015, 2016, 2017, 2018, 2019, 2020, 2021).

Sample sizes

Alzahrani [1] did not provide the final sample size for the analyses. We matched Alzahrani's initial sample size of 148,437 never-smokers. As mentioned, Alzahrani excluded participants who were former triers (n = 6,703). In Table 1, Alzahrani reports that there were 140,934 participants in his analyses: 139,967 never e-cigarette users and 1,237 current users. Alzahrani did not provide the final sample size after excluding participants who had missing data on age, sex, and health conditions; based on our re-analysis, we estimate that Alzahrani’s final sample was 135,991.

In our replication, we also excluded participants who had missing race/ethnicity and education. Our final sample was 132,603.

Statistical analysis

To replicate Alzahrani's model, we also used logistic regression to obtain the odds ratio (OR) of outcomes (heart attack, coronary heart disease, and stroke) among current e-cigarette users. In addition, we conducted the following sensitivity analyses to evaluate the stability of the association between current e-cigarette use and heart attack. First, we established a crude association without adjusting for any confounding factors, and then we added each factor to assess its effect on the OR for current e-cigarette use. The full model included age group, sex, race and ethnicity, levels of education, high cholesterol, diabetes, high blood pressure, overweight/obesity, region, and year. Next, we added former triers (n = 6,703), a group that Alzahrani [1] had completely excluded. Finally, we obtained the odds of having a heart attack by NHIS years, resulting in the exclusion of years 2015 and 2017 because of zero exposed cases.

## Results

Heart attack

We found only 12 current e-cigarette users reported heart attacks in the 2014-2021 NHIS dataset, which was described previously by Foxon et al. [2]. Table 1 lists the demographic characteristics and health conditions among the 12 exposed cases. The majority of these subjects were 50 years old or older, half reported high cholesterol levels and coronary heart disease, three-quarters reported diabetes, and all were overweight or obese. As noted, there were no e-cigarette-exposed heart attack cases in 2015 or 2017.

**Table 1:** Individual and health characteristics among current e-cigarette users who had heart attacks (n = 12) NH - non-Hispanic; HS - high school

NHIS year	Age	Sex	Race/ethnicity	Levels of education	High cholesterol	Diabetes	High blood pressure	Overweight/obese	Coronary heart disease	Region
2014	50	Men	NH Black	Some college	No	No	Yes	Yes	No	Northeast
2016	51	Women	NH White	≥ College	Yes	No	Yes	Yes	No	Northeast
2018	23	Men	NH White	Some college	No	Yes	No	Yes	No	South
2018	69	Women	NH White	Some college	No	No	Yes	Yes	No	West
2018	80	Men	Other races	Some college	Yes	No	Yes	Yes	Yes	South
2019	62	Women	Hispanic	Less than HS	Yes	Yes	Yes	Yes	No	South
2020	21	Men	NH White	HS	No	Yes	Yes	Yes	Yes	South
2020	30	Women	NH White	Some college	No	No	No	Yes	No	West
2021	54	Men	NH White	HS	Yes	No	No	Yes	Yes	West
2021	59	Men	NH White	Some college	Yes	No	Yes	Yes	Yes	West
2021	59	Men	NH White	HS	Yes	No	Yes	Yes	Yes	South
2021	75	Men	NH White	Some college	No	Yes	Yes	Yes	Yes	Northeast

Next, we replicated Table 1 of Alzahrani’s article (results are not shown). We successfully matched all of the results except overweight/obesity. We found 87,235 never users who were overweight or obese, compared with 90,164 reported by Alzahrani [1], while our number of current e-cigarette users who were overweight/obese matched Alzahrani (n = 686)). Notably, Alzahrani [1] reported a median age of 50 years for never users and 28 years for current users. However, those numbers are means, not medians. We found that the median age for current e-cigarette users was 24 years, compared with 50 years for never users. 

Despite the above discrepancy in the overweight/obese variable, we replicated Alzahrani’s ORs (Table 2).

**Table 2:** Replicated Alzahrani’s Table 2 95%CIs are presented in parentheses. *Our models excluded participants who had missing age, sex, high cholesterol, diabetes, hypertension, and BMI (n = 132,603).

	Model 1				Model 2			
	Alzahrani		Our model*		Alzahrani		Our model	
	OR	95%CI	OR	95%CI	OR	95%CI	OR	95%CI
E-cigarette use	2.30	(1.28, 4.13)	2.45	(1.36, 4.38)	2.62	(1.44, 4.77)	2.60	(1.43, 4.73)
Women	0.44	(0.41, 0.47)	0.44	(0.41, 0.47)	0.44	(0.41, 0.48)	0.45	(0.42,0.49)
Age	1.07	(1.07, 1.08)	1.08	(1.07, 1.08)	1.06	(1.06, 1.06)	1.06	(1.06, 1.07)
Hypertension					2.20	(2.01, 2.41)	2.20	(2.01, 2.41)
Diabetes mellitus					1.88	(1.73, 2.04)	1.85	(1.70,2.01)
High cholesterol					2.11	(1.95, 2.29)	2.09	(1.93, 2.26)
Obesity/overweight					1.16	(1.07, 1.27)	1.18	(1.08, 1.29)

Alternative explanations

We systematically investigated Alzahrani’s OR for heart attack among current e-cigarette users by using a stepwise adjustment of key variables. Table 3, Model 1, shows that the crude OR for current e-cigarette users was 0.42 (95%CI: 0.24-0.75), which was also found by Foxon et al. [2]. After adding age groups in Model 2, the direction of the relationship reversed, and the OR substantially increased to 3.00 (95%CI: 1.66-5.43). Additions of other variables in Models 3-7, (i.e., sex in Model 3, race/ethnicity in Model 4, levels of education in Model 5, health conditions in Model 6, and region and survey year in Model 7) slightly reduced the ORs to 2.48 (95%CI: 1.35-4.55). In Model 8, we omitted the age variable (while keeping all other covariates), and the OR dropped from 2.48 (95%CI: 1.35-4.45) in Model 7 to 0.80 (95%CI: 0.45-1.43). This demonstrates conclusively that the association between heart attack and e-cigarette use is largely moderated by age.

**Table 3:** The odds ratio for heart attack and current e-cigarette use* *All models excluded participants who had missing age, sex, race/ethnicity, education, high cholesterol, diabetes, hypertension, and BMI. 95%CIs are presented in parentheses. NH - non-Hispanic; HS - high school

Models	(1)	(2)	(3)	(4)	(5)	(6)	(7)	(8)
Current e-cigarette use	0.42	3.00	2.66	2.66	2.64	2.43	2.48	0.80
	(0.24, 0.75)	(1.66, 5.43)	(1.46, 4.83)	(1.46, 4.82)	(1.45, 4.78)	(1.33, 4.45)	(1.35, 4.55)	(0.45, 1.43)
Age groups								
18-24		Reference	Reference	Reference	Reference	Reference	Reference	Omitted
25-34		2.36	2.42	2.42	2.88	2.45	2.45	
		(1.21, 4.57)	(1.25, 4.69)	(1.24, 4.69)	(1.48, 5.59)	(1.26, 4.76)	(1.26, 4.75)	
35-44		4.55	4.67	4.66	5.49	3.71	3.71	
		(2.41, 8.59)	(2.47, 8.81)	(2.47, 8.78)	(2.91, 10.37)	(1.96, 7.01)	(1.96, 7.02)	
45-54		15.16	15.17	14.99	17.04	8.59	8.62	
		(8.25, 27.86)	(8.25, 27.87)	(8.16, 27.54)	(9.27, 31.34)	(4.66, 15.83)	(4.68, 15.88)	
55-64		35.58	36.14	35.49	39.28	15.32	15.47	
		(19.50, 64.91)	(19.81, 65.95)	(19.45, 64.78)	(21.51, 71.74)	(8.36, 28.07)	(8.44, 28.34)	
65+		85.34	93.91	92.39	96.49	31.01	31.46	
		(46.93, 155.16)	(51.64, 170.78)	(50.80, 168.06)	(53.01, 175.64)	(16.97, 56.70)	(17.21, 57.51)	
Sex	No	No	Yes	Yes	Yes	Yes	Yes	Yes
Race/Ethnicity	No	No	No	Yes	Yes	Yes	Yes	Yes
Levels of education	No	No	No	No	Yes	Yes	Yes	Yes
Health conditions	No	No	No	No	No	Yes	Yes	Yes
Regions	No	No	No	No	No	No	Yes	Yes
Survey years	No	No	No	No	No	No	Yes	Yes
Observations	132,603	132,603	132,603	132,603	132,603	132,603	132,603	132,603

In addition, we recategorized e-cigarette use as never users, current users, and former triers and applied the same models presented in Table 3. The inclusion of former triers (results are not shown) did not change current users’ ORs due to the fact that there were still only 12 heart attacks among current users.

Coronary heart disease and stroke

Table 4 presents the associations between current e-cigarette use and coronary heart disease and stroke. Model 1 for both outcomes shows the same pattern as heart attack, with respect to moderation by age. After adjusting for all other variables (i.e., age, sex, race/ethnicity, education, health conditions, region, and survey years) in Model 2, the ORs for coronary heart disease and stroke were not statistically significant; OR = 1.12 (95%CI: 0.58-2.17) for coronary heart disease and OR = 1.13 (95%CI: 0.55-2.29) for stroke. When we omitted age in Model 3, the ORs inverted again to 0.39 (95%CI: 0.21-0.73) for heart disease and 0.44 (95%CI: 0.22-0.88) for stroke.

**Table 4:** The odds ratio for coronary heart disease and stroke and current e-cigarette use* *All models excluded participants who had missing age, sex, race/ethnicity, education, high cholesterol, diabetes, hypertension, and BMI. 95%CIs are presented in parentheses. NH - non-Hispanic; HS - high school

	Coronary heart disease			Stroke		
Models	(1)	(2)	(3)	(1)	(2)	(3)
Current e-cigarette use	0.20	1.12	0.39	0.24	1.13	0.44
	(0.11, 0.38)	(0.58, 2.17)	(0.21, 0.73)	(0.12, 0.49)	(0.55, 2.29)	(0.22,0.88)
Age groups						
18-24		Reference	Omitted		Reference	Omitted
25-34		1.49			2.40	
		(0.94, 2.35)			(1.51, 3.79)	
35-44		1.94			3.66	
		(1.25, 3.00)			(2.35, 5.69)	
45-54		4.58			6.49	
		(3.01, 6.85)			(4.23, 9.94)	
55-64		9.44			9.28	
		(6.30, 14.13)			(6.08, 14.17)	
65+		22.39			17.91	
		(14.92, 33.29)			(11.77, 27.24)	
Sex	No	Yes	Yes	No	Yes	Yes
Race/Ethnicity	No	Yes	Yes	No	Yes	Yes
Levels of education	No	Yes	Yes	No	Yes	Yes
Health conditions	No	Yes	Yes	No	Yes	Yes
Region fixed effect	No	Yes	Yes	No	Yes	Yes
Year fixed effect	No	Yes	Yes	No	Yes	Yes
Observations	132,517	132,517	132,517	132,605	132,605	132,605

## Discussion

Despite the “correction” in Alzahrani's [3] discussion and conclusion, the author failed to document that his finding is based on only 12 heart attack cases, which is well below the number of cases required for stable logistic regression estimates (e.g., at least 20 cases per predictor) [12,13].

Therefore, Alzahrani’s results are statistically unstable, a point also raised by Foxon et al. [2]. In the current re-analysis, the instability is demonstrated by the fact that the crude OR for current e-cigarette use is 0.4. However, in our re-analysis, the addition of age to the model results in an inversion of the OR to 3.00. We have demonstrated the impact of age on Alzahrani’s full model in Model 8 of Table 3.

It is well-documented that cardiovascular disease is highly correlated with age [14], while e-cigarettes are most commonly used by young adults [15]. Alzahrani [1] adjusted for age in his model, but inappropriately as a continuous variable. In short, he assumed that one year increase in age has the same relationship with heart attack across the entire age spectrum (i.e., increasing from age 20 to 21 years has the same effect on heart attack as increasing age from age 59 to 60 years). Instead, we employed the appropriate adjustment of age groups in our model.

Nevertheless, Alzahrani [1,3] still made an inappropriate causal claim in the Discussion section. He wrote “These findings raise another concern regarding the future trend in the incidence of cardiovascular disease in young individuals who use e-cigarettes,” which implies causality. We emphasize that only two current e-cigarette users 18-24 years old reported heart attacks, one of which had two other risk factors, while the other had three (Table 1).

Furthermore, Alzahrani [1] did not evaluate effect modification by the other heart attack risk factors (overweight/obesity, diabetes, hypertension, high cholesterol). All of the cases among e-cigarette users had these risk factors. Attempting to control for these risk factors also adds to the instability of these findings, and thus the study could not assess the independent association between e-cig and heart attacks. That is, the study could not answer the question, “Is e-cigarette use, in itself and without the presence of other risk factors, associated with heart attacks?”.

Alzahrani's [1] finding also lacks biological plausibility. While the corrected article removed the conclusions about cardiovascular disease and stroke (which are unsupported by the finding), these are informative regarding the plausibility of Alzahrani’s findings; if e-cigarettes have a causal effect on cardiovascular outcomes, then this should also be evident for coronary heart disease and stroke. Our analysis found no statistically significant associations between e-cigarette use and these diseases, which raises further doubts about Alzahrani’s findings.

There are other major errors in Alzahrani’s article.

In Alzahrani’s correction, he correctly labeled his original citation by Bhatta et al. [16], which was later retracted by the journal editors [17] because it did not account for heart attacks that occurred before the first e-cigarette use, even though the information was available in the Population Assessment of Tobacco and Health dataset and after the temporality problem was raised in pre-publication peer review [18]. However, the current analysis likely suffers from the same flaw [19], as Alzahrani acknowledges in limitations “that it is not known when cardiovascular disease started relative to e-cigarette use in the study subjects.”

Inexplicably, the author omitted race and ethnicity from his recent study without justification; they are well-established to be important confounders for heart attack [20,21] and he included them in his previous study [22].

Another erroneous statement is made in limitations, where Alzahrani [1] claimed to have used propensity score analyses: “Propensity score analysis was used in this study to reduce confounding variables but cannot be used to establish causal inference.” However, logistic regression - not propensity score analysis - was used, as described in Alzahrani’s methods section, and confirmed in our current re-analysis. The impact of this inaccurate claim is to falsely minimize the severity of the limitation about unaccounted-for confounding.

In the same paragraph, Alzahrani [1] refers to the wrong dataset (BRFSS rather than NHIS), which again falls short of acceptable standards of scientific publishing.

Finally, it has been demonstrated by two of the current authors that cross-sectional datasets such as the NHIS, which do not contain information about the initiation or duration of exposure and timing of disease diagnosis, exaggerate associations and produce results that are unreliable [19]. For the multiple reasons detailed in this replication study, we recommend that Cureus editors consider retraction of Alzahrani’s article.

## Conclusions

The study by Alzahrani made erroneous claims and overstated the association between e-cigarettes and myocardial infarction. Our replication shows that the association is driven by age and there were no statistically significant associations with other cardiovascular diseases, coronary heart diseases, and stroke.
